# ADAM17, A Key Player of Cardiac Inflammation and Fibrosis in Heart Failure Development During Chronic Catecholamine Stress

**DOI:** 10.3389/fcell.2021.732952

**Published:** 2021-12-13

**Authors:** Joseph Adu-Amankwaah, Gabriel Komla Adzika, Adebayo Oluwafemi Adekunle, Marie Louise Ndzie Noah, Richard Mprah, Aisha Bushi, Nazma Akhter, Fei Huang, Yaxin Xu, Seyram Yao Adzraku, Iqra Nadeem, Hong Sun

**Affiliations:** ^1^ Department of Physiology, Xuzhou Medical University, Xuzhou, China; ^2^ Xuzhou Medical University, Xuzhou, China; ^3^ Key Laboratory of Bone Marrow Stem Cell, Department of Hematology, The Affiliated Hospital of Xuzhou Medical University, Xuzhou, China; ^4^ Department of Neurobiology and Anatomy, Xuzhou Medical University, Xuzhou, China

**Keywords:** heart failure, cardiac inflammation, cardiac fibrosis, ADAM17, metalloenzymes, pro-inflammatory cytokines, fibrotic factors

## Abstract

Heart failure development is characterized by persistent inflammation and progressive fibrosis owing to chronic catecholamine stress. In a chronic stress state, elevated catecholamines result in the overstimulation of beta-adrenergic receptors (βARs), specifically β2-AR coupling with Gαi protein. Gαi signaling increases the activation of receptor-stimulated p38 mitogen-activated-protein-kinases (p38 MAPKs) and extracellular signal-regulated kinases (ERKs). Phosphorylation by these kinases is a common way to positively regulate the catalytic activity of A Disintegrin and Metalloprotease 17 (ADAM17), a metalloprotease that has grown much attention in recent years and has emerged as a chief regulatory hub in inflammation, fibrosis, and immunity due to its vital proteolytic activity. ADAM17 cleaves and activates proinflammatory cytokines and fibrotic factors that enhance cardiac dysfunction via inflammation and fibrosis. However, there is limited information on the cardiovascular aspect of ADAM17, especially in heart failure. Hence, this concise review provides a comprehensive insight into the structure of ADAM17, how it is activated and regulated during chronic catecholamine stress in heart failure development. This review highlights the inflammatory and fibrotic roles of ADAM17’s substrates; Tumor Necrosis Factor α (TNFα), soluble interleukin-6 receptor (sIL-6R), and amphiregulin (AREG). Finally, how ADAM17-induced chronic inflammation and progressive fibrosis aggravate cardiac dysfunction is discussed.

**GRAPHICAL ABSTRACT:**
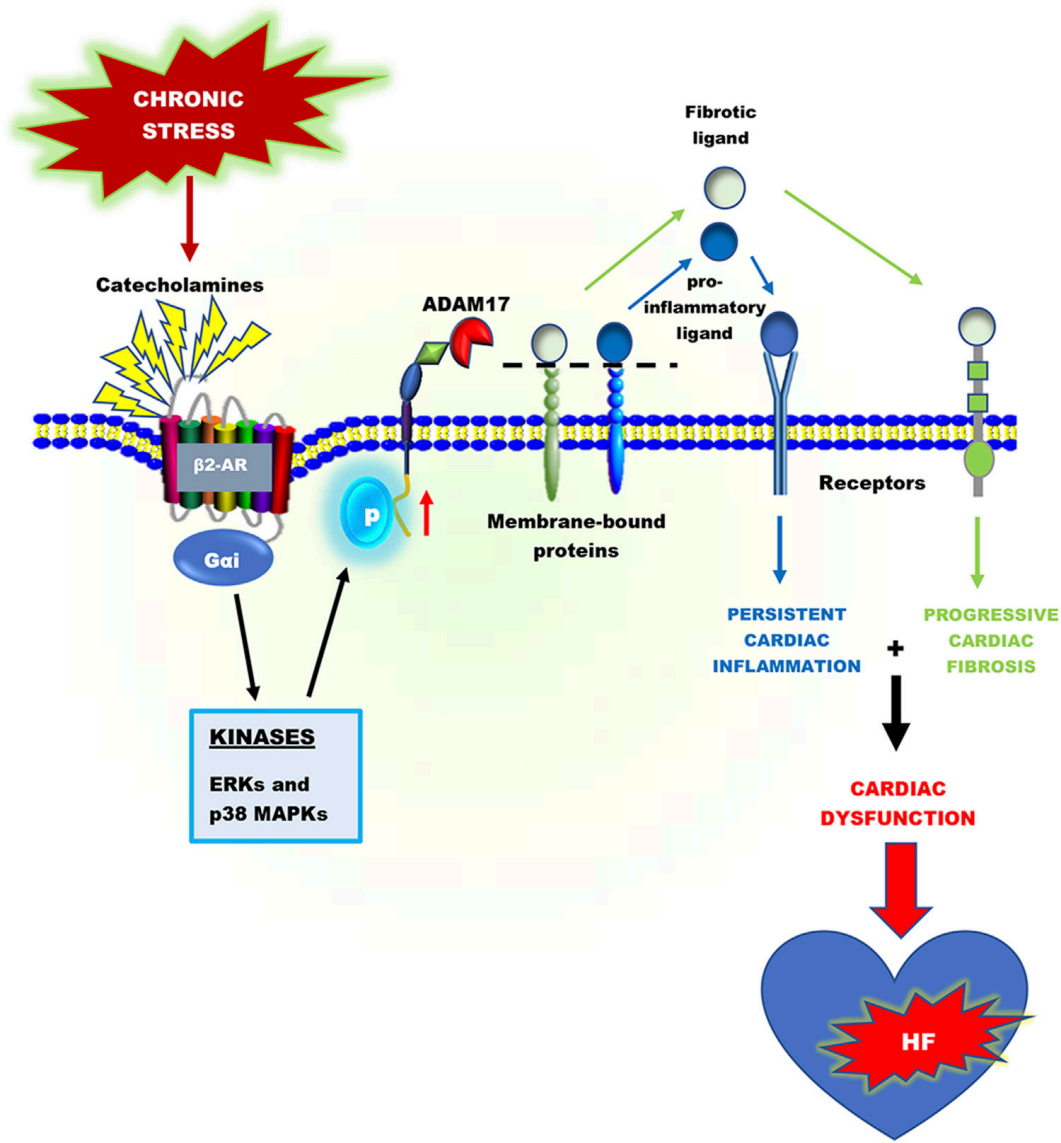


## Introduction

Heart failure (HF) is a serious clinical and public health issue that affects over 23 million people globally, resulting in significant mortality, morbidity, and healthcare expenditures (Ayoub et al., 2017; Orso et al., 2017; Frantz et al., 2018). Despite advances in understanding its pathophysiology and treatment, the prognosis of patients with HF remains poor. Approximately 2–17% of patients die during their first hospital stay, with over 50% of patients dying within 5 years (Ayoub et al., 2017).

Chronic stress-induced adverse cardiac remodeling and HF are generally associated with prolonged activation of proinflammatory responses (Adzika et al., 2021; Huo S. et al., 2021). In an inflammatory driven HF, the inflammatory responses are orchestrated by myosin and troponin (damage-associated molecular patterns (DAMPs)) released from necrotic cardiomyocytes. These cardiac antigens activate and induce the infiltration of neutrophils, macrophages, dendritic cells, as well as T and B cells into the myocardia (Lafuse et al., 2020; Adzika et al., 2021). Following cardiac injury, neutrophils and CD86^+^ macrophages are rapidly recruited to the injured area, where they initiate inflammatory responses with the goal of cleaning up dead cell debris. However, excessive accumulation and/or delayed switch from these proinflammatory cells infiltration to reparative inflammatory cells (such as CD206^+^ macrophages) has detrimental effects. By releasing reactive oxygen species, granular components, and proinflammatory mediators such as tumor necrosis factor-alpha (TNFα), soluble interleukin-6 receptor (sIL-6R), and CXC chemokine receptor 2 (CXCR2), neutrophils and macrophages contribute to adverse myocardial injury and remodeling (Adu-Amankwaah et al., 2021a; Ma, 2021). Additionally, the activation of T and B lymphocytes by dendritic cells has been shown to play crucial roles in myocardial inflammation (Santos-Zas et al., 2018; Santos-Zas et al., 2021). Ultimately, without timely resolution of these proinflammatory responses and initiates of reparative functions, genes encoding proinflammatory mediators and fibrotic factors are upregulated excessively (Epelman et al., 2014; Heidt et al., 2014; Adamo et al., 2020).

Proteolytic cleavage of transmembrane proteins is a vital post-translational modification that controls several transmembrane proteins’ biological function, including proinflammatory mediators and growth factors (Lichtenthaler et al., 2018; Düsterhöft et al., 2019). Amid the 560 proteases encoded in the human genome, A Disintegrin and Metalloprotease 17 (ADAM17) has grown much attention in recent years and has emerged as a chief regulatory hub in inflammation, fibrosis, and immunity due to its vital proteolytic activity (Düsterhöft et al., 2019). In immune and non-immune cells, ADAM17 cleaves a number of substrates, including ligands of the epidermal growth factor receptor (EGFR), adhesion molecules, proinflammatory cytokines, and chemokines and their receptors. Some of these substrates include amphiregulin (AREG), epigen, epiregulin, neuregulin, tomoegulin-2, transforming growth factor-alpha (TGF-α), heparin-binding epidermal growth factor (HB-EGF), TNFα, tumor necrosis factor β (TNFβ), the TNF receptors 1 and 2 (TNFR 1 and 2), and interlukin-6 receptor (IL-6R), CXCR2, collagen XVII, desmoglein-2 and nectin-4 (Black et al., 1997; Moss et al., 1997; Tellier et al., 2006; Reddy et al., 2009; Riethmueller et al., 2017; Kawai et al., 2021). According to Cabron et al. ADAM17 is a key regulator of soluble TNFα surface levels in proinflammatory macrophages and dendritic cells (Cabron et al., 2018). Additionally, sIL-6R and CXCR2 on human and mouse neutrophils surfaces are regulated by ADAM17 (Wright et al., 2014; Mishra et al., 2015).

In a physiological state, the expressions of ADAM17 in immune and non-immune cells are regulated by transcriptional and post-transcriptional factors, including nuclear factor kappa B (NF-κB) and Brahma-related gene 1 (BRG1). Furthermore, subcellular localization in the perinuclear region of cells has been shown to regulate ADAM17’s activity (Chemaly et al., 2017; Adu-Amankwaah et al., 2021a). Overexpression and chronic activation of ADAM17 can trigger excessive release of TNFα, sIL-6R, and CXCR2 on the surface of proinflammatory cells, which play crucial roles in the pathogeneses of several inflammatory diseases, including heart failure. Increased levels of TNFα, sIL-6R and CXCR2 have been implicated in immune cells (CD86^+^ macrophages, neutrophils, and dendritic cells) trafficking, migration, and activation as well as inducing excessive fibrosis, myocardial stiffness, and left ventricular diastolic dysfunction (Cumberbatch and Kimber, 1992; Bozkurt et al., 1998; Satoh et al., 2000; Russo et al., 2009; Jones et al., 2010; Anderson et al., 2013; Arokiasamy et al., 2017).

Several studies have shown that myocardial ADAM17, TNFα, and sIL-6R expressions in both mRNA and protein levels are higher in patients with cardiovascular diseases and complications, although ADAM17’s expression is downregulated in a normal state (Satoh et al., 1999; Damås et al., 2000; Satoh et al., 2000; Satoh et al., 2004; Anderson et al., 2013). Thus, establishing a positive correlation between ADAM17 and heart failure development. The increased expression of ADAM17, TNFα, and sIL-6R has a vital implication in aggravating cardiac dysfunction during heart failure development (Satoh et al., 1999; Satoh et al., 2000; Satoh et al., 2004; Adu-Amankwaah et al., 2021a). Additionally, pro-AREG, a bi-functional growth factor converted to its active form by ADAM17, is crucially involved in enhancing cardiac fibrosis and aggravating cardiac dysfunction (Liu et al., 2018). Besides inducing HF *via* facilitating hyperactive proinflammatory responses, ADAM17 has been implicated along with HB-EGF and betacellulin (BTC), and angiotensin-converting enzyme 2(ACE2) in causing congenital heart diseases and hypertensive-induced HF, respectively (Jackson et al., 2003; de Queiroz et al., 2015; Xu et al., 2017; Mukerjee et al., 2019). This comprehensive review provides an insight into the structure of ADAM17, how it is activated and regulated during chronic catecholamine stress in heart failure development. This review highlights the inflammatory and fibrotic roles of ADAM17’s substrates; TNFα, sIL-6R, and sAREG. Finally, how ADAM17-induced chronic inflammation and progressive fibrosis aggravate cardiac dysfunction is also discussed.

## A Disintegrin and Metalloprotease 17 and Other Related Metalloproteinases

### Overview

A disintegrin and metalloproteinases (ADAMs) consist of membrane-bound proteins that belong to a Zn^2+^-dependent protease superfamily. They are similar to other metalloenzymes, including matrix metalloproteinases (MMPs), meprins, and snake venom metalloproteinases (SVMP) (Calvete et al., 2007; Gooz, 2010). Physiologically, ADAMs and their related metalloenzymes are widely expressed in various body tissues and regulate diverse cellular activities, including cell migration, adhesion, proteolysis, and cellular signaling (Black et al., 1997; Jones et al., 2016). Hence, it is not astonishing that alterations in the expression or function of these proteases are implicated in several pathologies, including cancer, rheumatoid arthritis, kidney fibrosis, diabetes, Alzheimer’s disease, and cardiovascular diseases (Sandgren et al., 1990; Black et al., 1997; Satoh et al., 2000; Umemura et al., 2014; Kefaloyianni et al., 2016; Zhang et al., 2016; Kim et al., 2020; Shalaby et al., 2020; Adu-Amankwaah et al., 2021a). Increasing evidence suggests that various ADAMs and other related metalloenzymes play crucial roles in cardiovascular pathophysiology *via* the modulation of inflammation, angiogenesis, metabolism, cell proliferation, and cell migration (Adu-Amankwaah et al., 2021a; Kawai et al., 2021). Among the ADAMs identified so far (22 in humans, 34 in mice), ADAM8, 9, 10, 12, 17, 19 and closely related metalloenzymes including MMP2, MMP9, and meprin β are associated with cardiovascular conditions such as hypertension, atherosclerosis, aortic aneurysms, restenosis, acute coronary syndrome, cardiomyopathies and HF (Papazafiropoulou and Tentolouris, 2009; Broder and Becker-Pauly, 2013; Zhang et al., 2016; Adu-Amankwaah et al., 2021a; Kawai et al., 2021). According to Wichert et al., active meprin β is capable of inducing the proteolytic activities of ADAM9, 10, and 17 *via* specific prodomain cleavage (Wichert et al., 2019). The activation of MMP2 and MMP9 is part of the downstream signaling of ADAM10 and 17, which are closely related in structure and function (Xiao et al., 2012; Jones et al., 2013). While ADAM10’s expression may be important in cancer and neurological disorders, ADAM17 is primarily responsible for coordinating proinflammatory responses during stress. The various substrates of ADAMs have been extensively reviewed elsewhere (Kawai et al., 2021). Remarkably, several members of the ADAM family share the same substrates, and this nonspecific relationship between ADAMs and their substrates complicates and intrigues the physiology of ADAMs. However, the main focus of this review is to elucidate the mechanistic signaling pathways of ADAM17 in HF development during chronic stress.

**FIGURE 1:**
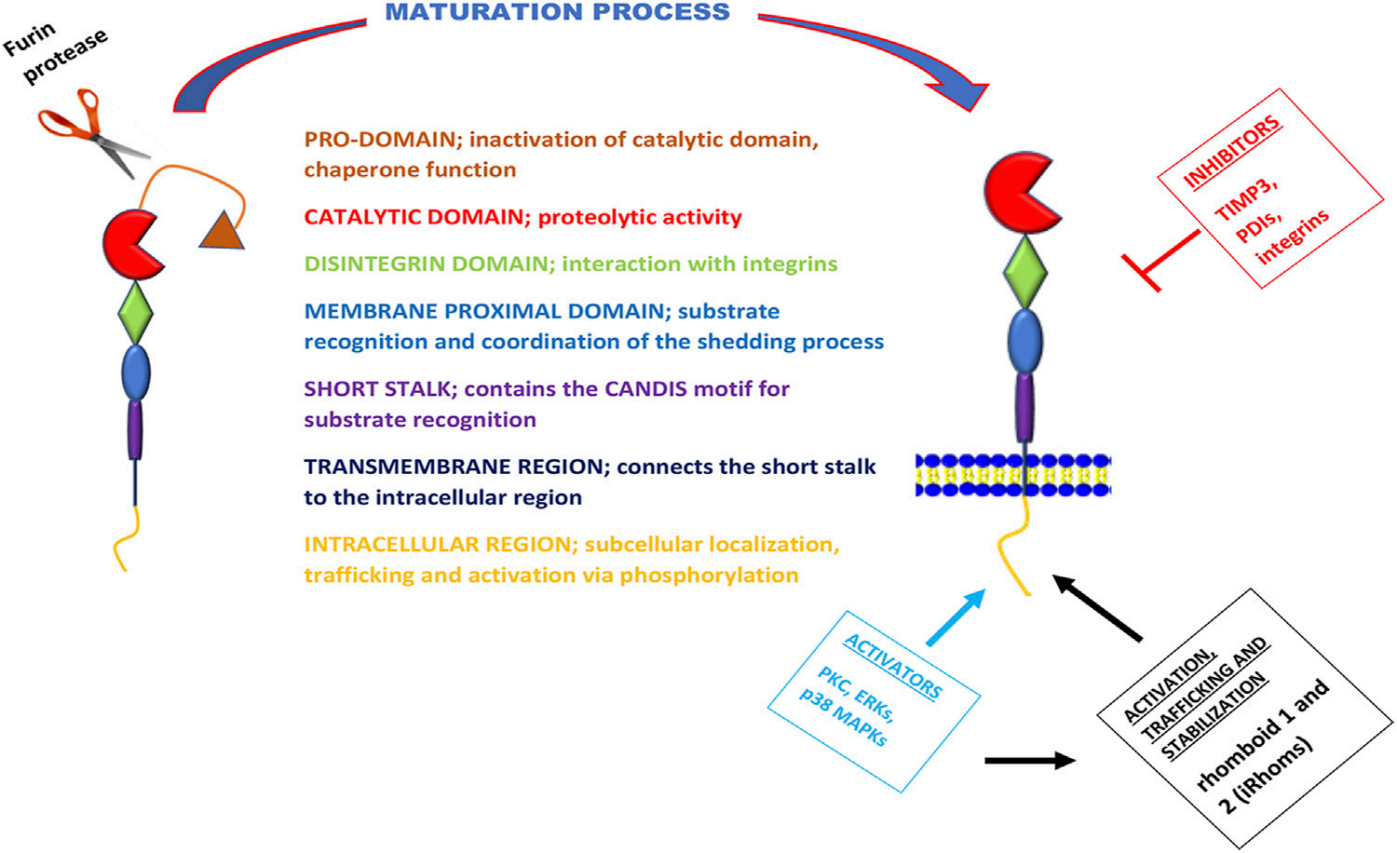
Schematic overview of the structure, function, maturation process, and regulation of ADAM17. The metalloprotease ADAM17 can be divided into seven domains with distinct functions, here separated by different colors. During maturation of ADAM17, the pro-domain is cleaved of by furin proteases. The activation of this metalloprotease is via its intracellular region by kinases; PKC, ERKs, and p38 MAPKs. These kinases are also known to phosphorylate and activate iRhoms for trafficking, stabilization, and cell surface expression of ADAM17. However, the inhibition of ADAM17 is mostly carried out by TIMP3, PDIs, and integrins.

ADAM17 was discovered in 1997 and named TACE (TNFα converting enzyme), as it was initially known as the protease that converts membrane-bound pro-TNFα (mTNFα) to a soluble form through its cleavage activity (Black et al., 1997; Moss et al., 1997). However, recent studies show that this protease is not only responsible for the liberation of soluble TNFα (sTNFα) but has a relatively broad spectrum of over 90 substrates (Black et al., 1997; Moss et al., 1997; Lammich et al., 1999; Garton et al., 2003; Reddy et al., 2009; Riethmueller et al., 2017). ADAM17 can be activated by intracellular kinases, which include phosphate kinase c (PKC), receptor-stimulated p38 mitogen-activated-protein-kinases (p38 MAPKs), and extracellular signal-regulated kinases (ERKs) (Bell and Gööz, 2010; Xu et al., 2012; Adu-Amankwaah et al., 2021a). These kinases can also phosphorylate and activate rhomboid 1 and 2 (also known as iRhoms or pseudoproteases) (Grieve et al., 2017), which are responsible for trafficking, stabilization as well as activation of ADAM17 (Adrain and Freeman, 2012; McIlwain et al., 2012; Maretzky et al., 2013; Li et al., 2015). Its inhibition is mostly done *via* tissue inhibitor of metalloproteinase 3 (TIMP3), integrins and protein disulfide isomerases (PDIs) (Düsterhöft et al., 2013; Düsterhöft et al., 2019; Park et al., 2019; Figure 1).

### A Disintegrin and Metalloprotease 17’s Structure

ADAM17 is a type-I transmembrane protein (Bode et al., 1993; Düsterhöft et al., 2019) with a similar class III snake venom metalloenzymes structure (Bode et al., 1993; Gooz, 2010). It comprises a prodomain, a catalytic domain, a disintegrin-like domain, a membrane-proximal domain (MPD), and a short stalk region, which together forms the extracellular part of the protease and are linked to an intracellular region (ICR) by a transmembrane part (Grötzinger et al., 2017). The catalytic domain possesses this metalloprotease’s proteolytic activity (Bode et al., 1993; Stöcker et al., 1995; Black et al., 1997); however, the preceding prodomain has chaperone-like functions that inhibit this catalytic activity, and it is cleaved off by furin proteases during the maturation of the protease (Schlöndorff et al., 2000). Though this cleavage step was primarily considered a prerequisite for the proteolytic activity of ADAM17, a study by Schwarz et al., revealed that ADAM17 was also active when cleavage by furin proteases was prevented by mutagenesis of the cleavage site (Schwarz et al., 2013). The disintegrin-like domain is needed for the interaction with integrins, a feature that ADAM17 shares with other ADAM family members (Bode et al., 1993; Düsterhöft et al., 2019). However, the membrane-proximal domain is only found in ADAM10 and ADAM17, but not the other family members (Stöcker et al., 1995; Grötzinger et al., 2017), and it is crucially involved in substrate recognition and coordination of the shedding process (Düsterhöft et al., 2013). The membrane-proximal domain is regulated by two disulfide bonds that are vulnerable to isomerization by PDI activity (Düsterhöft et al., 2013). The stalk region of ADAM17 contains the CANDIS motif (Conserved ADAM 17 Dynamic Interaction Sequence), which is located closer to the membrane-proximal domain near the plasma membrane and is vital for substrate recognition (Düsterhöft et al., 2014; Düsterhöft et al., 2019; Figure 1).

## Activation of A Disintegrin and Metalloprotease 17 During Chronic Stress

Chronic stress is a renowned risk factor for several cardiovascular diseases (Kivimäki and Steptoe, 2018). One of the central neural pathways activated by stress is the autonomic nervous system. During chronic stress, the sympathetic nervous system can be continuously activated, which results in elevated levels of catecholamines (epinephrine and norepinephrine) (Won and Kim, 2016). Epinephrine and norepinephrine function as hormones and neurotransmitters that maintain homeostasis *via* adrenergic receptors (ARs), including alpha-adrenergic receptors (α-ARs) and beta-adrenergic receptors (β-ARs). Studies have demonstrated the involvement of ADAM17 with α1-AR (Chen et al., 2006) and β-AR signaling (Zhu and Steinberg, 2021). β-ARs account for the majority of the total ARs in the heart (O’Connell et al., 2014), particularly in apical myocytes (Paur et al., 2012; Machuki et al., 2019) and cardiac non-myocytes such as endothelial and immune cells (Myagmar et al., 2017; Adzika et al., 2021). Hence, a continuous increase in the levels of epinephrine and norepinephrine can result in overstimulation of β-ARs (Machuki et al., 2019). Epinephrine is a more potent ligand for β-ARs compared to norepinephrine (Scanzano and Cosentino, 2015). β-ARs are 7-transmembrane, G-protein coupled receptors which are divided into four subtypes, namely; β1-AR, β2-AR, β3-AR, and β4-AR (Ahlquist, 1948; Bylund et al., 1994; Granneman, 2001). In the heart, the β1-AR, β2-AR, and β3-AR are all broadly expressed, with the β1-AR having the highest expression and the β3-AR having the lowest (Ahlquist, 1948; Madamanchi, 2007). The β4-AR is a low-affinity state of the β1-AR that is yet to be genetically and pharmacologically characterized (Granneman, 2001). The β2-AR and β3-AR can couple with Gαs or Gαi while β1-AR primarily couples with Gαs when activated (Machuki et al., 2019; Schena and Caplan, 2019). In physiological state, the activation of β2-AR and β3-AR couple with Gαs (Adzika et al., 2019; Machuki et al., 2019) and Gαi (Tchivileva et al., 2009; Schena and Caplan, 2019), respectively. Among these βARs, β2-AR is rarely depleted during stress, and it is also the most implicated in mediating signaling cascades in ventricular apical myocytes, cardiac endothelial and immune cells resulting in the initiation and progression of cardiovascular diseases (Paur et al., 2012; Adzika et al., 2019; Adu-Amankwaah et al., 2021a).

**FIGURE 2:**
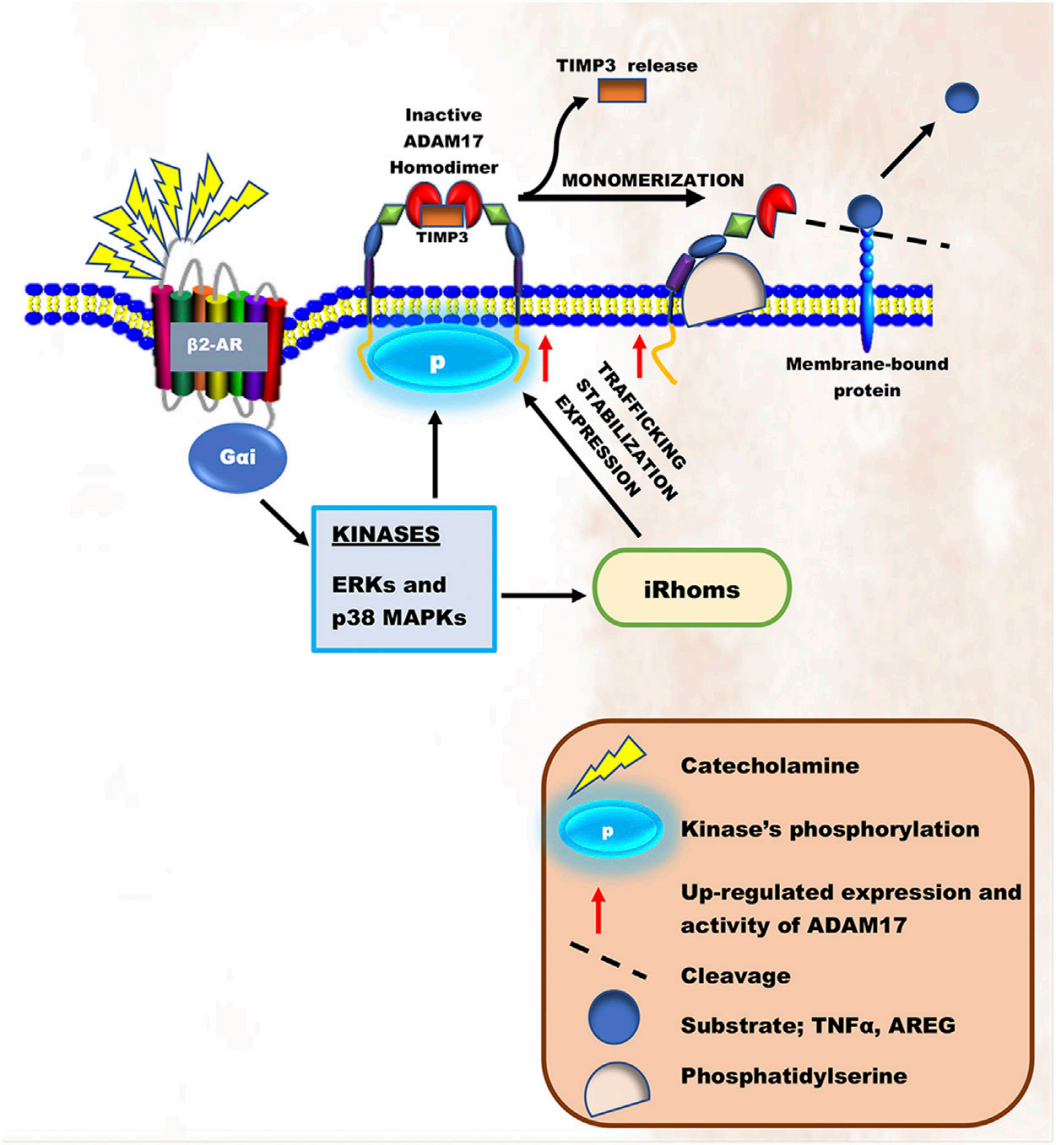
Schematic illustration of ADAM17’s activation during chronic catecholamine stress. Elevated catecholamines owning to chronic stress results in overstimulation of β2-ARs coupling with Gαi. Gαi signaling induces the activation of intracellular kinases, ERKs and p38 MAPKs. These kinases are known to either directly phosphorylate and activate ADAM17 or activate iRhoms responsible for trafficking, stabilization, and cell surface expression of ADAM17, thereby initiating its cleaving process.

In the heart, the overstimulation of β-ARs on ventricular apical myocytes, cardiac endothelial and immune cells due to elevated levels of circulating catecholamine desensitize β1-ARs (Bristow et al., 1990; Paur et al., 2012; Machuki et al., 2019). According to Zhu and Steinberg, the inactivation of β1-ARs and its irresponsiveness to catecholamines in cardiomyocytes during stressful events is *via* a mechanism involving N-terminal truncation at R^31^↓L^32^ by ADAM17 (Zhu and Steinberg, 2021). As such, β2-ARs coupling to Gαi is induced (Bristow et al., 1990; Paur et al., 2012; Machuki et al., 2019; Adzika et al., 2021). In short-terms, Gαi signaling increases via Akt/PI3K/p38 MAPKs/ERKs to prevent cardiac insult (Magocsi et al., 2007; Lajevic et al., 2011; Hou et al., 2018). Recent findings have suggested that the prolonged hyperstimulation of β2-ARs on ventricular apical myocytes and cardiac immune cells induces the bindings of β-arrestin-2 and G protein-coupled receptor kinases (GRKs) to scaffold non-canonical signaling that activates ERKs and p38 MAPKs activities maladaptively, ultimately resulting in HF (Shenoy et al., 2006; Paur et al., 2012; Adzika et al., 2019; Adzika et al., 2021). Intriguingly, ADAM17 initiates its adverse remodeling cascade upon being phosphorylated by these kinases directly and indirectly. For instance, during the maturation of ADAM17, ERK-dependent threonine 735 (Thr735) phosphorylation is vital for it to reach the secretory pathway (Díaz-Rodríguez et al., 2002; Chemaly et al., 2017). Also, indirect ERKs or p38 MAPKs phosphorylation at 14-3-3 binding sites on the N-terminal of iRhoms turns to induce ADAM17’s trafficking, stabilization, and cell surface expressions (Bell and Gööz, 2010; Adrain and Freeman, 2012; McIlwain et al., 2012; Xu et al., 2012; Li et al., 2015; Grieve et al., 2017). On the cell surface, mature ADAM17 proteins exist as inactive homodimers coupled to their inhibitor, TIMP3. However, activation of the ERK or p38 MAPK pathway directly phosphorylates Thr735 on the intracellular domain of ADAM17 and transforms it from a dimer structure into an active monomer structure liberating it from TIMP3 (Xu et al., 2012; Chemaly et al., 2017). Following monomerization, ADAM17 then binds to the phosphatidylserine exposure at the outer leaflet of the cell membrane via its MPD and CANDIS, thereby initiating its cleaving process (Gooz, 2010; Figure 2).

## A Disintegrin and Metalloprotease 17 in Cardiac Inflammation

ADAM17 plays a key role in cardiac inflammation, as it can cleave and activate several proinflammatory cytokines and their receptors. The most prominent examples include the cytokine TNFα, the TNFR 1 and 2, and the IL-6R (Black et al., 1997; Moss et al., 1997; Tellier et al., 2006; Reddy et al., 2009; Riethmueller et al., 2017).

### Tumor Necrosis Factor α and its Receptors

**FIGURE 3:**
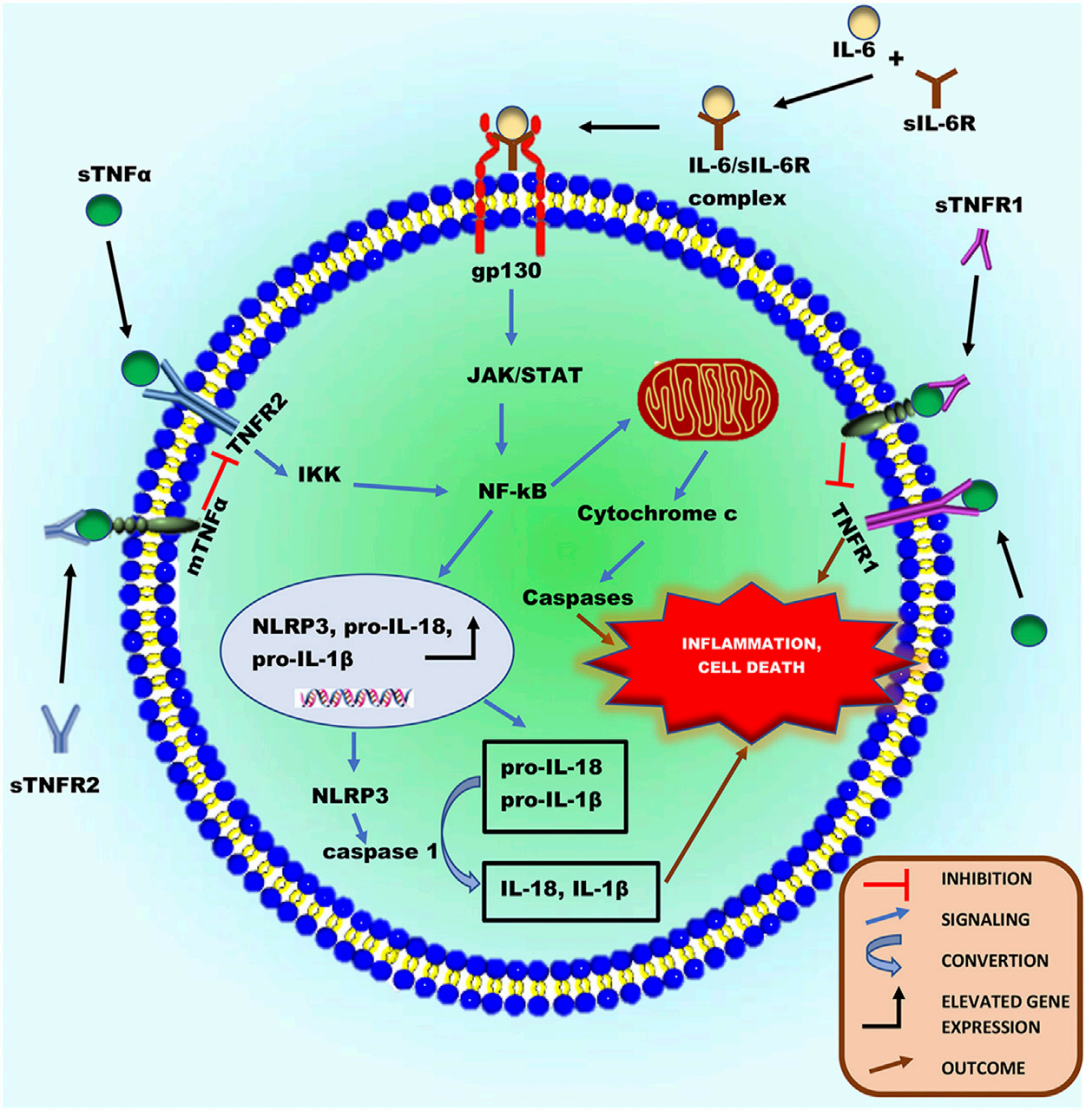
Schematic illustration of the inflammatory roles of ADAM17’s substrates, sTNFα and sIL-6R in a cardiac cell during chronic catecholamine stress. Following the proteolytic processing of ADAM17, sTNFα and IL-6 can bind to TNFR1/2 and sIL-6R, respectively activating downstream signaling cascades. Activated TNFR1 can directly induce inflammation and programmed cell death. The activation of TNFR2 can cause it to interact with the IκB kinase (IKK) complex resulting in the phosphorylation of IκB, thereby activating NF-κB. Also, the IL-6/sIL-6R complex formed from IL-6 binding to sIL-6R can directly activate the ubiquitously expressed glycoprotein-130 (gp130), thereby activating NF-κB. Activated NF-κB can either migrates into the nucleus or mitochondria. In the nucleus, it encodes genes of proinflammatory cytokines (pro-IL-18 and pro-IL-1β) and NLRP3, increasing their protein expression. NLRP3 inflammasome can activate caspase 1, which in turn cleaves pro- IL-1β and pro- IL-18 to release their soluble forms, to induce necrosis and inflammation in cardiac cells. Additionally, in the mitochondria, activated NF-κB can stimulate intrinsic apoptotic pathways via releasing cytochrome c, which triggers caspase cascades resulting in programmed cell death and inflammation.

The cytokine TNFα is a typical type-II transmembrane protein that belongs to the TNF superfamily (Düsterhöft et al., 2019). It is expressed as a membrane-bound protein, activated by the cleavage process of ADAM17 to release sTNFα (Black et al., 1997; Moss et al., 1997). The majority of the proinflammatory activities of TNFα are attributed to its soluble form. This cytokine activation can signal via two different receptors, TNFR1 and TNFR2 (Defer et al., 2007; Salmeri et al., 2015), expressed on cardiac myocytes (Defer et al., 2007). Interestingly, TNFR1 and 2 can also be cleaved from the surface of cells by ADAM17, and the resulting soluble TNFR (sTNFR) ectodomains retain their ability to bind mTNFα and therefore act as antagonistic decoy receptors (Rego et al., 2013; Figure 3). Besides their function as decoy receptors, sTNFR1 and 2 can also perform a very different biological function by binding to mTNFα on the cell surface and inducing signals within TNFα-expressing cells. This concept is known as “reverse signaling,” which is common among other TNF family members (Juhász et al., 2013). Although both TNFR1/2 can bind to the ligand sTNFα, the intracellular signaling cascades triggered, and the biological responses are markedly different (Düsterhöft et al., 2019). Most importantly, the intracellular region of TNFR1 contains a death domain capable of inducing direct programmed cell death when activated, which is absent in the intracellular region of TNFR2 (Düsterhöft et al., 2019). The binding of sTNFα to TNFR2 can result in the activation of nuclear factor kappa B (NF-κB) (Albensi, 2019; Adu-Amankwaah et al., 2021a), which is also expressed in myocytes, cardiac endothelial, and immune cells (Li et al., 2020). The NF-κB complex exists in an inactive state in the cytoplasm (Ghosh et al., 1998; Albensi, 2019). However, activation of TNFR2 can interact with the IκB kinase (IKK) complex resulting in the phosphorylation of IκB, subsequently causing IκB ubiquitination and degradation, leading to the activation of NF-κB dimer (Li and Karin, 2000; Israël, 2010). When activated, it then migrates into the nucleus (Sen and Smale, 2010) or mitochondria (Bottero et al., 2001; Cogswell et al., 2003). In the nucleus, it encodes genes of proinflammatory cytokines (pro-IL-18 and pro-IL-1β) and NLR family pyrin domain containing 3 (NLRP3) (Sen and Smale, 2010; Albensi, 2019). NLRP3 is an intracellular sensor that identifies a wide range of environmental irritants, microbial motifs, and endogenous danger signals, resulting in the formation and activation of the NLRP3 inflammasome. Activation of the inflammasome triggers caspase 1, which in turn, cleaves pro- IL-1β and pro- IL-18 to release their soluble forms (Bauernfeind et al., 2009; Xing et al., 2017; Swanson et al., 2019), thereby inducing necrosis and inflammation in cardiac cells (Li et al., 2018). Studies show that activated NF-κB can stimulate the intrinsic apoptotic pathway in the mitochondria *via* releasing cytochrome c, which triggers caspase cascades resulting in programmed cell death (Liu et al., 2004; Albensi, 2019; Adu-Amankwaah et al., 2021a; Figure 3). Irrefutably, increased levels of TNFα in the stress state has been linked to the pathophysiology of heart failure development in various clinical investigations (Ferrari et al., 1995; De Biase et al., 2003; Dunlay et al., 2008) and animal models (Bozkurt et al., 1998; Bryant et al., 1998; Moe et al., 2004; Guggilam et al., 2007; Adzika et al., 2021; Hou H. et al., 2021). For instance, a study carried out by Bryant et al. reveals that cardiac myocytes’ overproduction of TNFα is sufficient to cause heart failure, implying that this cytokine plays a causative role in the development of heart failure (Bryant et al., 1998). In an experimental heart failure model, *in vivo* TNFα inhibition reduced cardiac mitochondrial dysfunction, oxidative stress, and apoptosis (Moe et al., 2004). Additionally, in heart failure rats, TNF-alpha inhibition reduced chronic catecholamine-induced stress in the paraventricular nucleus and ameliorated cardiac function (Guggilam et al., 2007).

### IL-6 and its Receptor

**FIGURE 4:**
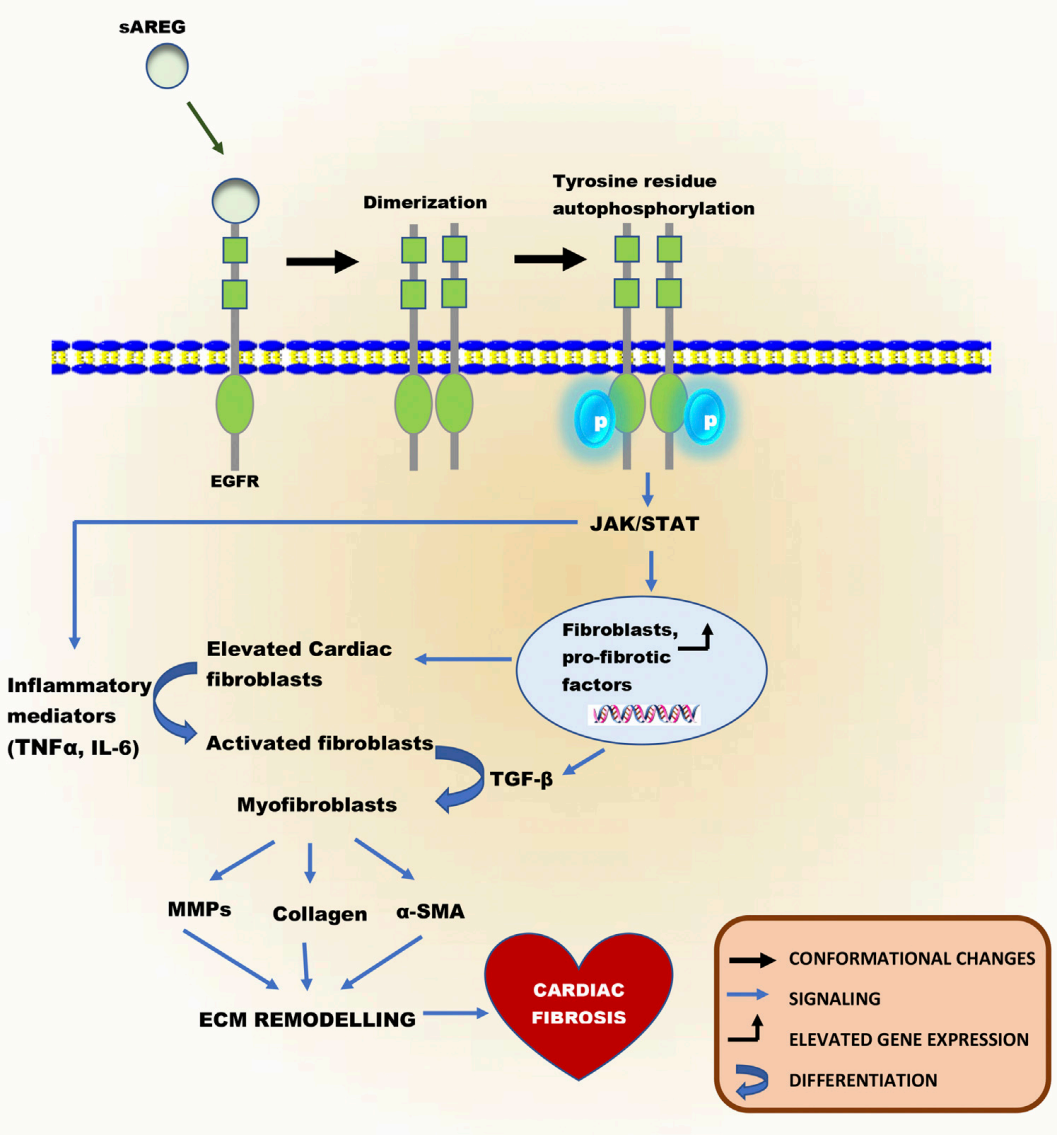
Schematic illustration of the fibrotic role of sAREG, a substrate of ADAM17. After the proteolytic process of ADAM17, sAREG can activate EGFR, which is widely expressed on cardiac cells. The binding of sAREG to EGFR causes the receptor to undergo a conformational change known as “Dimerization,” resulting in homo- or heterodimers formation. This precedes an intracellular domain activation in its tyrosine residues by phosphorylation, promoting these same residues’ autophosphorylation in their homolog. Autophosphorylation of EGFR can activate it to induce the JAK/STAT pathway, leading to an increase in gene and protein expression of fibroblasts and pro-fibrotic factors such as TGF-β. Elevated fibroblasts can result in fibroblast activation, both mechanically by altered activation patterns and chemically by inflammatory mediators. Activated fibroblasts are transformed into myofibroblasts by TGF-β. Myofibroblasts are not present in normal cardiac tissue unless during cardiac injury and can induce pathological ECM remodeling, which characterizes cardiac fibrosis via the expression of α-SMA, collagen synthesis, and secretion of MMPs.

IL-6 is a pleiotropic cytokine released in response to perturbations in homeostasis (Fontes et al., 2015). This cytokine has well-defined pro- and anti-inflammatory properties when activated. Interestingly, its receptor, IL-6R, can be cleaved by ADAM17 (Riethmueller et al., 2017; Garbers et al., 2018). The properties of IL-6 are determined by its stimulation and signaling processes. Thus, acute stimulation of IL-6 is mostly protective, while its chronic response causes long-term signaling leading to inflammation and autoimmunity (Fontes et al., 2015). Signaling *via* the membrane-bound IL-6 receptor (IL-6R) termed “classic signaling,” can only occur on cell types that express surface IL-6R, including hepatocytes and certain leukocytes’ subpopulations such as neutrophils (Wolf et al., 2014). However, signaling via soluble forms of the IL-6R, called IL-6 trans-signaling, can occur on all body cells since the IL-6/sIL-6R complex can directly bind to and activate the ubiquitously expressed glycoprotein-130 (gp130) without the need of a membrane-bound IL-6R (Wolf et al., 2014; Düsterhöft et al., 2019). IL-6 trans-signaling accounts mainly for the cytokine’s proinflammatory properties (Fontes et al., 2015; Düsterhöft et al., 2019). The glycoprotein-130 (gp130) receptor is widely expressed in mammals, including the developing and adult hearts (Podewski et al., 2003). In physiological state, activation of gp130 in the heart by IL-6 type cytokines induces signaling through three main pathways: 1) the Janus kinase/signal transducer and activator of transcription (JAK/STAT) pathway, 2) the phosphatidylinositol-3-kinase-dependent (PI3K)/Akt pathway and 3) the Ras/mitogen-activated protein kinase (MAPK) and extracellular signal-regulated kinase (ERK) signaling pathway (Podewski et al., 2003; Fischer and Hilfiker-Kleiner, 2008). These pathways have been demonstrated to play vital roles in cardiac development and protection (Yajima et al., 2006; Fischer and Hilfiker-Kleiner, 2008). However, in a chronic stress state characterized by elevated IL-6 and sIL-6R, continuous activation of gp130 in the heart can induce cardiac inflammation *via* gp130/JAK/STAT pathway (Podewski et al., 2003). This pathway can promote NF-κB activation, resulting in the release of proinflammatory cytokines, formation, and activation of inflammasomes, which mediate cell death and cardiac inflammation (Fischer and Hilfiker-Kleiner, 2008; Figure 4). Undeniably, it has been revealed that increased levels of gp130 proteins and IL-6 cytokines are strong predictive markers for morbidity and mortality in patients with HF (Fischer and Hilfiker-Kleiner, 2008). According to Ritschel et al., elevated levels of circulating sIL-6R and IL-6 were linked to future cardiovascular events and mortality in patients, implying that the IL-6 signaling pathway plays an essential role in the development of HF (Ritschel et al., 2016). Studies have also reported that the local gp130 receptor system in myocytes is altered in failing human hearts (Podewski et al., 2003; Fischer and Hilfiker-Kleiner, 2008). Furthermore, many animal studies have demonstrated that in a stress state, upregulated levels of IL-6 in myocardia enhance the development of heart failure while its inhibition improves cardiac function. (Lai et al., 2012; Zhao et al., 2016; Adzika et al., 2021; Hou H. et al., 2021; Huo S. et al., 2021).

Currently, ADAM17 has not been directly associated with the regulation of T and B cells functions in the myocardia; however, these immune cells secret TNFα, which is keenly regulated by ADAM17 (Opata et al., 2013; Yang et al., 2013; Adu-Amankwaah et al., 2021a). Also, ADAM17’s targets on T and B cells have been implicated in their migration, differentiation, and effector functions (Link et al., 2017). Typically, Marczynska et al. demonstrated that the costimulatory ligand, ICOS ligand (ICOSL), is preferentially downregulated on the surface of B cells in an ADAM17-dependent way, despite the fact that recombinant ADAM17 does not proteolyze it *in vitro* (Marczynska et al., 2014). Therefore, it can be speculated that ADAM17 might directly regulate T and B cells’ functions in the myocardia during inflammation.

## A Disintegrin and Metalloprotease 17 in Cardiac Fibrosis

ADAM17 is known to activate amphiregulin (AREG) via its proteolytic cleavage activity (Liu et al., 2018). AREG is synthesized as a type-I transmembrane protein (pro-AREG) that can engage in juxtracrine signaling on adjacent cells (Berasain and Avila, 2014). Alternatively, after proteolytic processing, the release of soluble AREG (sAREG) can act as an autocrine or paracrine factor (Berasain and Avila, 2014). sAREG is a ligand of the EGFR (Berasain and Avila, 2014; Liu et al., 2018), widely expressed on cardiac myocytes and fibroblasts (Berasain and Avila, 2014; Liu et al., 2018). In a physiological state, activation of EGFR in the heart induces major intracellular signaling cascades governing fibroblasts proliferation, migration, and collagen synthesis. However, prolonged activation of EGFR in a chronic stress state characterized by continuous elevation of sAREG can enhance cardiac fibroblast activation, proliferation, differentiation to myofibroblast, migration, and collagen synthesis (Liu et al., 2018). The binding of sAREG to EGFR, causes the receptor to undergo a conformational change inducing homo- or heterodimers formation (Dawson et al., 2005; Rayego-Mateos et al., 2018). This precedes an intracellular domain activation in its tyrosine residues by phosphorylation, promoting these same residues’ autophosphorylation in their homolog. Autophosphorylation of EGFR can activate it to induce the JAK/STAT pathway (Dawson et al., 2005; Rayego-Mateos et al., 2018). This signaling pathway plays a vital role in transducing stress and growth signals in the heart during cardiac fibrosis (Wagner and Siddiqui, 2012). The activation of the JAK/STAT pathway can increase the gene expression of fibroblasts and pro-fibrotic factors such as transforming growth factor-beta (TGF-β) (Wang et al., 2002). Physiologically, cardiac fibroblasts are responsible for the homeostasis of the extracellular matrix (ECM), which provides a structural scaffold for cardiomyocytes, distributes mechanical forces through the cardiac tissue, and mediates electrical conduction (Travers et al., 2016). However, elevated fibroblasts can result in fibroblast activation, both mechanically by altered activation patterns and chemically by inflammatory mediators (Wang et al., 2002). Notably, elevated TNFα and IL-6 secretions from macrophages, T and B lymphocytes during chronic inflammation also contributes to the aggravation of cardiac fibrosis as these cytokines stimulate fibroblast proliferation, differentiation to myofibroblast, and their migration (Wang et al., 2002; Adekunle et al., 2021). Also, TGF-β plays a vital role in ECM remodeling, cell mobility, and modulation of immune function. Increased levels of it are crucial in cell differentiation and proliferation of activated fibroblasts to myofibroblasts (Baum and Duffy, 2011). Myofibroblasts are not present in normal cardiac tissue unless during cardiac injury (Manabe et al., 2002). Myofibroblasts can induce pathological ECM remodeling (Manabe et al., 2002) *via* the expression of smooth muscle alpha-actin (α-SMA) (Sousa et al., 2007), collagen synthesis, and secretion of MMPs (Manabe et al., 2002). MMPs are responsible for the breakdown of the extracellular matrix in many diseases (Liu et al., 2006). Chronic secretion of MMPs in the heart leads to the degradation of collagen and elastin into peptide fragments resulting in elevated collagen deposition in the ECM, leading to scar formation. Although the formation of fibrotic scar tissue is an adaptive way of maintaining the structural integrity and pressure-generating capacity of the heart, myofibroblast persistence due to chronic stress can eventually result in the development of adverse changes in ventricular structure and compliance, which characterizes cardiac fibrosis (Liu et al., 2006; Figure 4). Intriguingly, ADAM17 upregulation does enhance the secretions of the aforementioned cytokine from both innate and adaptive immune cells either via direct or indirect cascade to cause maladaptive interstitial fibrosis.

## Synergy of A Disintegrin and Metalloprotease 17-Induced Inflammation and Fibrosis in Heart Failure Development

HF development is characterized by a progressive condition associated with left ventricular (LV) systolic or diastolic dysfunction resulting in insufficient oxygen and nutrient supply to peripheral organs. It can exist in two main forms, namely, HF with preserved ejection fraction (HFpEF) and HF with reduced ejection fraction (HFrEF) (Van Linthout and Tschöpe, 2017; Adu-Amankwaah et al., 2021b). HFpEF is accompanied by diastolic dysfunction characterized by impaired ventricle relaxation and filling, increased ventricle stiffness, and elevated filling pressure to respond to pressure overload (Adekunle et al., 2021). On the flip side, HFrEF is associated with systolic dysfunction characterized by impaired left ventricular contractility, resulting in a reduced ejection fraction (Tanai and Frantz, 2015). Cardiac inflammation and fibrosis play a central role in HF development (Liu et al., 2018). Both can trigger HF development under several conditions, ranging from acute stress to chronic catecholamine stress.

The outcome of inflammation and fibrosis can contribute to the pathogenesis of the two main forms of HF. Although elevated serum concentrations of proinflammatory cytokines and fibrotic factors are common in both forms of HF, the pathomechanisms involved in each are different. For HFpEF, studies reveal that the outcome of chronic inflammation leads to progressive fibrosis, which eventually results in LV hypertrophy (Manabe et al., 2002; Salles et al., 2007; Wynn, 2008). Excessive increase in ECM components and cross-linking during LV hypertrophy can induce myocardial stiffness, thereby triggering HFpEF-specific, characterized by concentric cardiac remodeling and LV diastolic dysfunction (Paulus and Tschöpe, 2013). For example, collagen I which stiffens the myocardia, accounts for about 80% of the total collagen in the myocardia and increases most during LV hypertrophy (Barison et al., 2015). In addition, excessive cross-linking during LV hypertrophy stiffens the collagen matrix, making it more difficult to be broken down by proteinases (Travers et al., 2016). According to Hieda et al., increased myocardial stiffness is frequently observed in patients with HFpEF (Hieda et al., 2020). Regarding HFrEF, excessive cardiomyocyte death preceding necrosis or apoptosis due to persistent cardiac inflammation can result in cardiac atrophy. Continued loss of cardiac tissue can induce systolic dysfunction leading to HFrEF-specific, characterized by eccentric cardiac remodeling and dysfunction (Van Linthout and Tschöpe, 2017). Undeniably, several large studies have reported that patients with HFrEF characterized by systolic dysfunction have elevated serum levels of proinflammatory cytokines such as TNFα, IL-6, and IL-1β (Torre-Amione et al., 1996; Rauchhaus et al., 2000; Deswal et al., 2001).

## Conclusion and Future Perspectives

ADAM17 is widely expressed by several mammalian cells. Evidence suggests that the number of identified substrates of this metalloprotease keeps increasing, implying that ADAM17 may play a central role in regulating several physiological and pathophysiological processes. Hence, its implicated in several human diseases as such heart failure is expected. Although, current drug treatments and the subsequent use of recognized medications have reduced mortality and hospitalization rate, particularly in HF patients with reduced ejection fraction (Berliner and Bauersachs, 2017), HF remain a major clinical and public health concern since it affects more than 23 million worldwide (Ayoub et al., 2017; Orso et al., 2017; Frantz et al., 2018); hence there is still a lot to discover about this condition which will serve as a key in establishing specific treatment and management guidelines to significantly reduced the rate of HF. This comprehensive review has provided extensive insight into the mechanisms underlying catecholamine-induced HF. Therefore, therapeutic prospects for the treatment and management of catecholamine-induced HF should also target the inhibition of ADAM17 and/or antagonize its activation and activities.

For decades, ADAM17 has been the subject of intense research. Since its identification as the tumor necrosis factor convertase, it has been an important therapeutic target, particularly in the setting of inflammatory diseases. Nonetheless, developing medications that target ADAM17 has proven more difficult than anticipated. This is owing to ADAM17’s multifunctionality, which includes the release of approximately 90 other substrates aside from tumor necrosis factor (TNF), as well as its structural similarities to other metalloproteinases (Calligaris et al., 2021). The most promising targets of ADAM17 (without any significant physiological consequences) appear to be inhibiting its phosphorylation by ERKs, p38 MAPKs, iRhom1, and iRhom2. These regulators are vital for trafficking, stabilization, and activation of ADAM17 (Bell and Gööz, 2010; Adrain and Freeman, 2012; McIlwain et al., 2012; Xu et al., 2012; Li et al., 2015; Grieve et al., 2017). Usage of pharmacologic agents capable of impeding ADAM17 phosphorylation by ERKs and p38 MAPKs may be an attractive potential target for downregulating ADAM17’s proteolytic activity. Also, it is well-known that iRhom2 is mainly expressed in proinflammatory immune cells, such as macrophages and neutrophils (Adrain and Freeman, 2012), whereas iRhom1 is predominantly expressed in non-immune cells (Issuree et al., 2013; Chemaly et al., 2017). Hence, it is tempting to hypothesize that inhibition of iRhom2 would aid in the downregulation of ADAM17 with no effects on non-immune cells. iRhom1 activities may then compensate for the iRhom2 blockade. Also, the inhibition of ADAM17 could be achieved by injecting its natural inhibitors (TIMP3, PDIs, and integrins). Notably, injection of TIMP3 has been shown to prevent heart failure post-myocardial infarction (Martz, 2014; Takawale et al., 2017; Chintalgattu et al., 2018). PDIs can also interact directly with ADAM17’s MPD, which catalyzes the isomerization of two disulfide bridges, lowering ADAM17’s activity (Willems et al., 2010). Furthermore, the binding of integrin α5β1 to ADAM17 *via* its disintegrin domain, according to Bax et al., inhibited its activity by altering its mediated cell adhesion and migration (Bax et al., 2004). Besides its natural inhibitors, miRNAs such as miR-124 (Sun et al., 2013), miR-145 (Doberstein et al., 2013), miR-152 (Su et al., 2014), and miR-326 (Cai et al., 2015) have been shown to suppress ADAM17 expression and limit substrate release by binding directly to the ADAM17 3′-UTR.

The modulation of ADAM17 is key in ameliorating cardiac function *via* attenuation of myocardial inflammation during chronic catecholamine stress. Thus, minimizing the levels of TNFα and other proinflammatory cytokines is necessary for the heart’s normal function; hence, inhibiting ADAM17 which facilitates the activities of these cytokines, might enhance cardiac health or delay the progression of its pathological remodeling into HF.
